# County Hospitals

**Published:** 1916-05

**Authors:** 


### County Hospitals.

The county hospital movement should spread in Texas until every county in the State without a public hospital has one. Such institutions are needed not only by the poor, but by those who are able to pay all their bills. The homes of the country are not equipped for the proper care of the sick, and protracted illness is a heavy drain upon every member of the household in which the sick person is. Comfortable hospitals with reasonable charges are not only a convenience but they should be considered as an economic necessity. However, they should be so conducted that they will not prove a burden instead of a help to the people in which they are built and operated. It is bad enough to be sick and to have to meet the reasonable bills incident thereto, but it is little short of criminal to extort onerous fees and charges from the unfortunate.

